# Essential amino acids and branched-chain amino acids are associated with skeletal muscle and inflammatory parameters in older age

**DOI:** 10.1007/s10522-025-10206-1

**Published:** 2025-03-06

**Authors:** Ching Wah Donna Li, Catrin Herpich, Ulrike Haß, Bastian Kochlik, Daniela Weber, Tilman Grune, Kristina Norman

**Affiliations:** 1 Department of Nutrition and Gerontology, German Institute of Human Nutrition Potsdam-Rehbrücke, Nuthetal, Germany 14558; 2 Faculty of Health Science Brandenburg, Department of Rehabilitation Medicine, University of Potsdam, Potsdam, Germany 14476; 3 Department of Food Safety, Federal Institute for Risk Assessment, Berlin, Germany 10589; 4 Department of Geriatrics and Medical Gerontology, Charité - Unniversitätsmedizin Berlin, Berlin, Germany 13347; 5 Department of Molecular Toxicology, German Institute of Human Nutrition Potsdam-Rehbruecke, Nuthetal, Germany 14558; 6 Institute of Nutritional Science, University of Potsdam, Potsdam, Germany; 7 German Center for Cardiovascular Research (DZHK), Partner Site Berlin, Berlin, Germany

**Keywords:** Aging, Muscle health, Amino acids, Inflammation, Sarcopenia, Metabolism

## Abstract

Aging is associated with a decline in muscle mass and function, increasing the risk of adverse health outcomes. Amino acid profiling has emerged as a potential tool for assessing skeletal muscle health. This study examines the associations between fasting plasma amino acids, muscle function, and inflammation in healthy older and young adults. Data from 131 participants (101 older adults, 71.5±4.9 years; 30 young adults, 25.5±3.9 years) were analyzed. Skeletal muscle mass was assessed using bioimpedance analysis, and hand grip strength was measured with a dynamometer. Plasma amino acids, kynurenine, and inflammatory markers (CRP, IL-6) were quantified using ultraperformance liquid chromatography with tandem mass spectrometry and commercial immunosorbent assays, respectively. Older adults exhibited lower levels of glutamic acid, isoleucine, leucine, phenylalanine, kynurenine, and kynurenine-to-tryptophan (KYN:TRP) ratio compared to younger individuals (all p<0.05). In older adults, branched-chain and essential amino acids correlated positively with skeletal muscle index (SMI) and hand grip strength, whereas in young adults, only glutamic acid, proline, and KYN:TRP showed positive associations with SMI (all p<0.05). CRP and IL-6 were associated with several amino acids in older adults but not in younger individuals. These findings suggest that age-related shifts in amino acid profiles may reflect underlying changes in muscle metabolism and function, highlighting their potential as early indicators of muscle decline.

## Introduction

Aging is marked by a gradual and inevitable decrease in muscle strength and mass, which may result in sarcopenia. This condition leads to functional limitations in mobility and an increased risk of adverse health outcomes, posing a serious problem in the aging society (Papadopoulou 2020; Morley et al. 2014). The trajectory of this decrease is highly individual and hinges on factors including physical activity status, age, sex, and metabolic health, highlighting the broader complexity of aging as a non-uniform and multifaceted process (Rattan 2024).

Amino acid profiling may serve as a promising tool to assess skeletal muscle health in older individuals (Calvani et al. 2020). The body's free amino acid pool is distributed in the plasma and cellular spaces, where they are influenced by metabolic variations, diet, lifestyle, and genetic factors (Wagenmakers 1998; Scalbert et al. 2009). Despite representing only 2% of the total amino acids in the body, these circulating amino acids play a pivotal role in skeletal muscle plasticity and are involved in various biological processes, including inflammation, insulin sensitivity, and redox balance (Zhenyukh et al. 2017; Yoon 2016). Their role has been linked to age-related muscle deterioration and the metabolic changes observed in sarcopenia. As aging heterogeneity remains a key knowledge gap in geroscience, understanding age-related changes in amino acids may help unravel its causes and consequences (Rattan 2024).

Skeletal muscle serves as the primary source of circulating amino acids during the post-absorptive state, with levels proportionate to muscle mass (Hagen et al. 2023; Felig 1975). This relationship highlights the importance of circulating amino acid concentrations as indicators of amino acid homeostasis within muscle tissues (Hagen et al. 2023). Importantly, distinct amino acid profiles have been identified in older individuals with obesity (Takashina et al. 2016), sarcopenia (Calvani et al. 2018), frailty (Calvani et al. 2018), and insulin resistance (Calvani et al. 2020). Older adults with physical frailty and sarcopenia show a different profile compared to their healthy counterparts, suggesting that metabolic changes are reflected in levels of circulating amino acids.

The skeletal muscle mass index (SMI) along with hand grip strength have been widely utilized in the screening of sarcopenia and assessment of muscle health (Kawakami et al. 2022; VanItallie et al. 1990; Janssen et al. 2000). In older Japanese women, plasma branched chain amino acids (BCAA) and essential amino acids (EAA) were positively associated with hand grip strength (Yamada et al. 2018). This association suggests that amino acid levels could serve as potential indicators of muscle strength.

Given the intricate relationship between circulating amino acids, functional muscle qualities, and their potential modulation by inflammatory factors, the primary objective of the present study is to investigate the associations between fasting free AA, inflammatory factors and muscle mass and function in older compared to younger individuals. This analysis aims to identify an amino acid pattern in healthy older adults to inform future biomarkers for muscle health deterioration.

## Methods

### Participants

This is a secondary analysis of two studies published elsewhere (Herpich et al. 2021; Hass et al. 2022). We used data from 131 healthy, community-dwelling young and old adults (n = 64 older women, n = 37 older men, n = 21 young women, n = 9 young men) from the Berlin-Brandenburg region in this analysis. All participants gave written informed consent. The studies received ethical approval from the University of Potsdam ethics committee and were registered at DRKS00018995 and DRKS00017090, respectively, and carried out in accordance with the Declaration of Helsinki.

### Dietary intake

Dietary intake was derived from 24 h dietary recalls and assessed with the German software EBISpro version 2011, (Dr. J. Erhardt, Stuttgart, Germany) which is based on the German food code.

### Anthropometric measures

Weight (kg), height (cm) were measured according to standard protocol and body mass index (BMI) was calculated as weight/height^2^ (kg/m^2^). Body composition was assessed with Bioimpedance Analyzer (Quantum/S Akern, Florence, Italy; with resolution on impedance components: resistance (Rz) 1% and reactance (Xc) 2% according to the manufacturer). Skeletal muscle mass was calculated (Janssen et al. 2000) and also expressed as skeletal muscle mass index (SMI) (kg/m^2^).

### Hand grip strength

The hand grip strength of the dominant hand was measured using a dynamometer (Jamar, Preston Bissell Health Care Co., Jackson, MI, USA) with the arm at an right angle and the elbow by the side of the body. The participant was told to squeeze the dynamometer with maximum effort for 5 s. The representative value was defined as the highest achieved value using the dominant hand.

### Analytic procedures

Blood samples were obtained between 7:30 am and 9:00 am after an overnight fast and stored at − 80 °C until analysis. Twelve amino acids and the tryptophan derivative kynurenine were measured in the plasma with ultraperformance liquid chromatography with tandem mass spectrometry (UPLC-MS/MS). Plasma samples (10 µL) were mixed with 40 µL of an internal standard solution (62.5 µM labeled amino acids in 90% acetonitrile) and vortexed. Protein was precipitated by storing samples at − 20 °C for 10 min and then centrifuging at 30,000×*g* and 4 °C for 10 min. A 2 µL aliquot of the supernatant was injected into the UPLC-MS/MS system. Amino acids were separated using an ACQUITY UPLC BEH Amide column (2.1 mm × 100 mm, 1.7 µm) with a VanGuard BEH Amide pre-column (2.1 mm × 5 mm, 1.7 µm). The gradient elution involved mobile phase A (acetonitrile/MilliQ water with ammonium formate and formic acid) and mobile phase B (MilliQ water with ammonium formate). The total runtime was 10 min with a flow rate of 0.4 mL/min. Samples were kept at 6 °C in the autosampler, and the column temperature was set at 35 °C. The Xevo TQ-MS mass spectrometer was operated in ESI-positive mode, with a desolvation temperature of 600 °C, desolvation gas flow of 600 L/h, source capillary voltage at 0.5 kV, cone gas flow at 150 L/h, and nebulizer gas pressure at 7.0 bar. Plasma free amino acids are shown in absolute but also normalized to skeletal muscle mass. Moreover, the following sum scores were calculated for amino acids: BCAA (isoleucine, leucine and valine), EAA (Isoleucine, leucine, phenylalanine, threonine, tryptophan, methionine, valine), non-essential amino acids (NEAA: tyrosine, alanine, glutamic acid, glycine, proline). The following ratios were calculated EAA:NEAA and KYN:TRP. Serum C-reactive protein (CRP) levels (mg/L) (intra-assay CV: 5.1–6.8%, inter-assay CV: 11.6–14.3%, apDia, Turnhout, Belgium) and interleukin-6 (IL-6) concentrations (pg/mL) were assessed using commercial immunosorbent assays (Intra-assay CV: 4.2–5.1%, inter-assay CV: 4.7–5.0%; BioVendor, Brno, Czech Republic).

### Statistical analysis

Data were expressed either as the mean and standard deviation (SD) when data was normally distributed or as medians and interquartile range (IQR) when not normally distributed. Differences in amino acid concentration between young and old were evaluated with the Mann–Whitney *U* test. Associations between amino acid concentrations and muscle and inflammatory parameters, and protein intake were determined with Spearman’s (rho) correlations. Results were considered statistically significant when the P value was below 0.05.

## Results

Characteristics of 131 study participants are displayed in Table 1. As anticipated, older adults had higher BMI, but lower SMI and grip strength. Inflammatory parameters did not differ between age groups.

**Table 1:** Characteristics of study population

	Young (n = 30)	Old (n = 101)	p value
Subject characteristics			
Female (n)	21	64	< 0.001
Age (years)	25.5 ± 3.9	71.5 ± 4.9	< 0.001
Height (m)	1.70 ± 0.1	1.66 ± 0.1	< 0.001
Weight (kg)	71.2 ± 15.2	74.9 ± 14.1	0.202
BMI (kg/m^2^)	23.5 ± 3.4	26.9 ± 4.1	< 0.001
PA level (METs)	1.7 ± 0.4	1.3 ± 0.5	< 0.001
Number of drugs (n)	0 ± .1	2 ± 2	< 0.001
Muscle mass and strength			
SMI	8.8 ± 1.3	8.3 ± 1.6	< 0.001
Handgrip strength (kg)	39.6 ± 12.4	31.6 ± 8.6	< 0.001
Dietary intake			
Total calories	2109.1 ± 701.1	1830.1 ± 516.8	0.009
Protein (g/day)	82.2 ± 44.4	69.7 ± 20.3	0.016
Fat (g/day)	81.9 ± 33.1	83.9 ± 28.9	0.378
Carbohydrate (g/day)	251.4 ± 88.9	184.5 ± 60.6	< 0.001
Inflammatory factors			
CRP (mg/L)	1.31 ± 2.1	2.14 ± 2.7	0.062
IL-6 (pg/mL)	3.67 ± 1.6	4.60 ± 5.6	0.188

*BMI* body mass index, *PA level* physical activity level, *METs* metabolic equivalents, *SMI* skeletal muscle index, *CRP* C-reactive protein, IL-6 interleukin-6

**Fig. 1:**
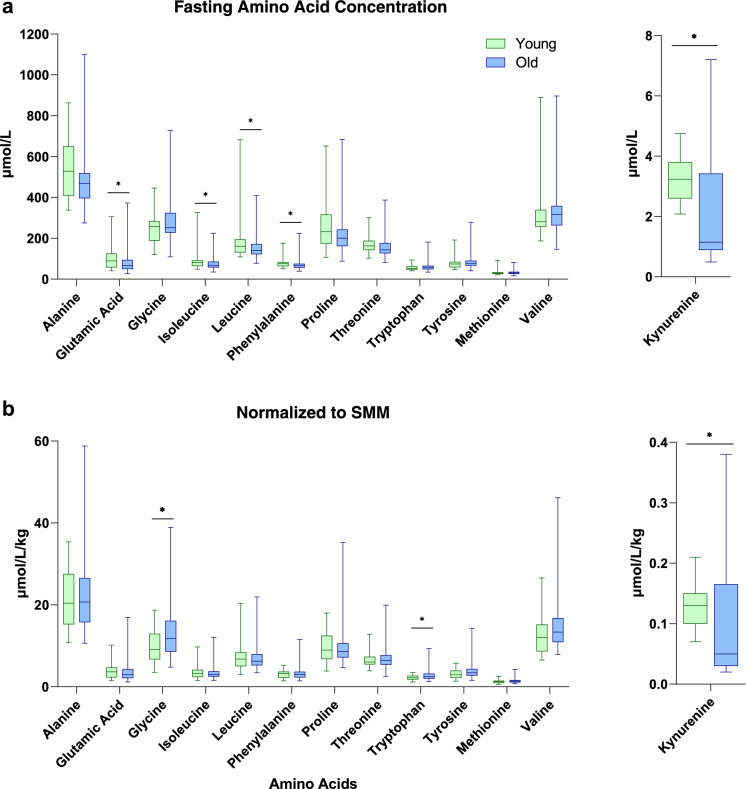
Box-and-whisker plot of plasma amino acid concentrations in young and old adults. Results were expressed as (a) absolute concentration, and (b) absolute concentrations normalized to total SMM. *P<0.05

Fasting amino acid concentrations and kynurenine were altered in older adults. Significant differences were observed for glutamic acid, isoleucine, leucine, phenylalanine, and kynurenine as well as KYN:TRP (Fig. 1A and Table 2). Amino acid concentrations were normalized to skeletal muscle mass (Fig. 1B). After normalization to skeletal muscle mass, glycine, tryptophan, and kynurenine were different between groups. Further analysis that kynurenine (rho = 0.347, p < 0.001) and KYN:TRP (rho = 0.337, p < 0.001) were both positively associated with age within the old study participants, albeit no association was seen with tryptophan (rho = 0.010, p = 0.917).

**Table 2:** Plasma levels of grouped amino acids and ratios

Amino Acids	Young (n = 30)	Old (n = 101)	p value
BCAA (µmol/L)	536.95 (465.82–621.39)	530.30 (441.09–609.65)	0.681
EAA (µmol/L)	684.73 (605.29–747.66)	698.64 (582.85–781.07)	0.952
NEAA (µmol/L)	1213.72 (998.62–1409.84)	1078.62 (954.24–1219.38)	0.082
EAA:NEAA	0.60 (0.50–0.70)	0.61 (0.56–0.68)	0.697
KYN:TRP	0.05 (0.048–0.06)	0.02 (0.01–0.06)	< 0.001

*BCAA* branched-chain amino acids, *EAA* essential amino acids, *NEAA*: non-essential amino acids, *EAA:NEAA* ratio of essential to non-essential amino acids, *KYN:TRP* kynurenine to tryptophan ratio

**Fig. 2:**
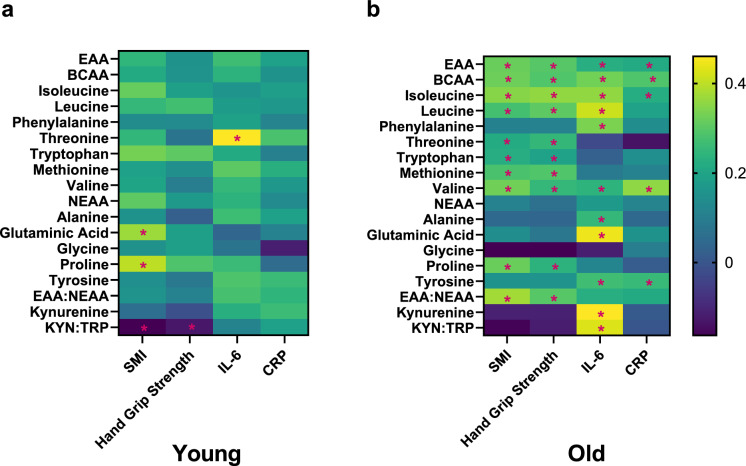
Association of Amino acid and metabolite concentrations with skeletal muscle and inflammatory markers. (a) Associations in young adults and (b) in older adults. *P<0.05

Amino acids were associated with muscle parameters. In the young group, glutamic acid, proline, and KYN:TRP correlated with SMI (p < 0.05). Only KYN:TRP correlated with hand grip strength (Fig. 2A).

In the old group, SMI was positively correlated with isoleucine, leucine, threonine, tryptophan, methionine, valine, BCAAs, EAA, EAA:NEAA and KYN:TRP. As well, hand grip strength was positively correlated with isoleucine, leucine, threonine, tryptophan, methionine, valine, proline, BCAAs, EAA and EAA:NEAA (Fig. 2B).

Neither CRP nor IL-6 were correlated with the muscle parameters. While IL-6 only correlated with threonine in the young group (Fig. 2A), significant positive correlations were found between IL-6 and alanine, glutamic acid, isoleucine, leucine, phenylalanine, tyrosine, valine, kynurenine, BCAA, EAAs, as well as KYN:TRP in the old study participants. Moreover, a positive correlation was seen between CRP and isoleucine, valine, tyrosine, BCAA and EAAs in the old study participants. (Fig. 2B). Protein intake per kg body weight correlated negatively with glutamine (r = − 0.368, p < 0.001), phenylalanine (r = − 0.217, p = 0.029), and tyrosine (r = − 0.276, p = 0.005) in older adults but showed no associations in the young (data not shown).

## Discussion

In this study, fasting plasma amino acids were characterized and associated with muscle and inflammatory parameters in young and old adults. The results show that (1) Fasting plasma amino acid concentrations differed between old and young, (2) EAAs and BCAAs are associated with muscle parameters in older adults and (3) EAAs and BCAAs were associated with inflammatory factors in older adults.

### Fasting amino acids concentrations differ with age

The present study observed lower amino acid concentrations in older adults. This is in general agreement with previous results (Milan et al. 2015; Foroumandi et al. 2018) and may be attributed to various factors such as a decline in protein synthesis and breakdown (Winterer et al. 1976), loss of lean body mass (Novak 1972), alterations in hormonal control (i.e. insulin, growth hormone) (Elahi et al. 1982), decreased protein intake (Timmerman and Volpi 2008), oxidative stress (Bala et al. 2021), or inflammaging (Sorgdrager et al. 2019a). These findings reinforce the broader notion that aging is not a linear or uniform process, but rather one influenced by species-specific and individual variations in metabolic regulation (Rattan 2024).

Under normal conditions, following an overnight fast when muscle protein net balance is negative, amino acids arise solely from skeletal muscle and the gut and is directed to the liver for gluconeogenesis or ketogenesis (Hagen et al. 2023). Thus, circulating amino acid concentrations are affected proportionally by protein catabolism in skeletal muscle and amount of muscle mass (Jourdan et al. 2012). This may differ in older age as muscle protein metabolism becomes compromised.

To test whether the lower amino acid concentrations observed in older adults were due to loss in skeletal muscle, we adjusted amino acid concentrations to skeletal muscle mass. Age-related differences disappear when adjusted for skeletal muscle mass, suggesting that the loss of skeletal muscle is responsible for the decrease in the amino acid concentrations, especially BCAAs. This finding supports the metabolic and regulatory role of skeletal muscle in BCAA levels. Further research is needed to conclude whether skeletal muscle acts as a source or sink for observed changes in circulating amino acid levels.

### Essential AAs are associated with muscle function parameters

In our study, plasma EAAs which encompass BCAAs were positively associated with SMI assessed with BIA. EAAs has been shown to be associated with muscle cross sectional area (Layman and Walker 2006) as well as with fat-free mass. EAAs, particularly BCAAs, are key stimuli for anabolism in skeletal muscle. This relationship is consistent with the finding that BCAA concentration after an overnight fast is proportional to muscle mass in the body (Layman and Walker 2006). The positive associations between EAAs including BCAAs and SMI in our analysis provide further evidence of BCAAs as plasma indicators of muscle status in older adults.

Plasma concentrations of leucine, isoleucine and phenylalanine were lower in older adults compared to younger adults. All three amino acids are EAAs. This age-related reduction may suggest reduced muscle quality. Yamada et al. (2018) demonstrated that lower concentrations of these amino acids in older women with dynapenia and sarcopenia correlated with reduced grip strength. In their previous study where skeletal muscle characteristics were assessed with ultrasound, muscle quality was shown to be lower in this cohort (Yamada et al. 2017). The authors suggested that lower plasma AA concentrations are related to qualitative changes in the muscle. Our study supports these findings, showing a positive association between plasma EAAs, including BCAAs, and grip strength.

### BCAAs are positively correlated with inflammatory factors

BCAAs were positively correlated with IL-6 in our study, however, only in older adults. Given the role of systemic inflammation in sarcopenia (Antuna et al. 2022), it is possible that BCAAs influence inflammatory pathways, though the direction and causality of this relationship remain unclear. Zhenyukh and colleagues (Zhenyukh et al. 2017) found that high concentrations of BCAAs (~ 10 mmol) can directly trigger oxidative stress (reactive oxygen species production) and NFkB-induced overexpression of pro-inflammatory genes (IL-6) through Peripheral Blood Mononuclear Cells activation. During inactivity or muscle atrophy, the role of NFkB is unequivocal (Li et al. 2008) and enhanced muscle protein catabolism overnight likely involves NFkB action. The liberation of muscle BCAAs may activate NFkB to further perpetuate oxidative stress. Furthermore, accumulated age-related oxidative damage to macromolecules (Beckman and Ames 1998) changes their secretory profile (e.g., mammalian target of rapamycin (mTOR) complexes) (Chandrasekaran et al. 2017). As protein synthesis is mainly mediated by the signaling cascade involving mTOR, these changes may alter anabolic regulation (Cuervo et al. 2005; Droge 2004). Furthermore, oxidative stress leads to increased NFkB activity which further exacerbates low-grade inflammation (Chandrasekaran et al. 2017).

### Age and inflammation impacts tryptophan metabolism

We observed an increase and decrease in tryptophan and kynurenine, respectively, from young to old. Tryptophan metabolism has been found to be diminished in older adults compared to their younger counterpart (Jasbi et al. 2023). This likely impact kynurenine conversion and leads to an accumulation of tryptophan. However, direct comparison between young and old adults of plasma concentrations of tryptophan and its metabolite have yielded mixed results. A previous study reported a decrease in tryptophan and increase in kynurenine levels with age (Trepci et al. 2020). In young and old Japanese men and women, old women had higher levels of tryptophan than both young men and women, and kynurenine levels were higher in young women and old men than in young men and old women (Masuda et al. 2019). With another Japanese study population, lower levels of tryptophan in old women and lower levels of kynurenine in young men were found (Tomioka et al. 2020). While it is evident that tryptophan metabolism is altered with age, it is difficult to conclude a direct difference between age groups.

Interestingly, within the older group, both kynurenine and KYN:TRP were positively correlated with age, but this was not true for tryptophan. Previous reports indicate an age-related decrease in circulating tryptophan and an increase in kynurenine in middle-aged (Capuron et al. 2011; Theofylaktopoulou et al. 2013; Ramos-Chavez et al. 2018). While our kynurenine results are consistent with these findings, our tryptophan results are not. One possible explanation for this inconsistency could be the use of plasma samples, as tryptophan is partially bound to proteins in the plasma (Cynober 2002).

In the present analysis, kynurenine and KYN:TRP were associated with IL-6 levels in the older group Kynurenine conversion is catalyzed by tryptophan 2,3‐dioxygenase (TDO) and indoleamine 2,3‐dioxygenase (IDO), with the latter activated by inflammatory factors such as IL-6, contributing to kynurenine buildup. (Sorgdrager et al. 2019b; Baumgartner et al. 2019). Considering the potential inflammation-mediated pathway, the KYN:TRP ratio is referred to as a biomarker of inflammaging and strongly associated with aging in humans (Sorgdrager et al. 2019a). This is in coherence with previous studies where tryptophan metabolites were associated with circulating IL-6 in older adults (Lustgarten and Fielding 2017; Al Saedi et al. 2022). Elevated tryptophan metabolites like kynurenine may increase oxidative stress and inflammation. (Hamrick and Isales 2020).

Even after adjustment of AA concentration to skeletal muscle mass, older muscle had lower levels of kynurenine but higher levels of tryptophan per unit of skeletal muscle, indicating these differences are not due to the age-related loss of skeletal muscle mass. TDO is the primary enzyme for the metabolism of the tryptophan pathway (Badawy 2017). In rats, TDO activity has been shown to decrease in the brain, liver and kidney with age (Braidy et al. 2011). A decrease in TDO activity may prohibit the conversion to kynurenine. Kynurenine may also accumulate in the skeletal muscle by direct uptake (Martin et al. 2020), ultimately clearing it from circulation. Vastus lateralis biopsies have shown that older sedentary adults have higher baseline levels of both kynurenine and tryptophan compared to young active adults (Hinkley et al. 2023), supporting this theory.

After normalization of amino acid concentration to skeletal muscle mass, glycine was one of the two amino acid that appeared to differ between young and old adults. Interestingly, plasma concentrations of glycine were found to be higher in the old group. Glycine is used to produce tripeptide glutathione (GSH), an antioxidant (Forman et al. 2009). As GSH levels have been reported to decline with age (Sekhar et al. 2011), therefore higher circulating levels might be reflective of decreased GSH synthesis by the liver.

### Limitations

The study has several limitations that should be acknowledged. Firstly, only cross-sectional data was analyzed, which does not capture potential changes over time. Therefore, only a snapshot of the amino acid profile following an overnight fast is captured, preventing the drawing of causal inferences. Additionally, bioimpedance analysis is not a direct measurement of body composition and its validity relies on the use of appropriate equations to assess body compartments such as skeletal muscle mass (Norman et al. 2012). Also, results are less reliable in disease with altered hydration. We used an equation which was validated with MRI in young and old healthy individuals but cannot preclude that biological factors affect the validity of results. Furthermore, the young group was a smaller sample and consisted mostly of females, therefore limiting strong comparisons and sex-stratified analyses.

## Conclusion

In summary, the study provides valuable insights into the association between basal plasma amino acids, muscle function, and inflammatory parameters in young and old adults. We have shown that age-associated lower levels of BCAAs, certain EAAs, and NEAAs are likely due to reduced skeletal muscle mass, highlighting the role of skeletal muscle as a key metabolic reservoir. However, the absence of major disruptions between the two age groups suggests that these metabolic shifts may serve as early indicators of dysfunction, emerging before more pronounced declines in muscle mass and function occur.

## Data Availability

No datasets were generated or analysed during the current study.
